# Di-μ_1,1_-azido-bis­[(2-{1-[2-(isopropyl­amino)ethyl­imino]eth­yl}phenolato)copper(II)]

**DOI:** 10.1107/S1600536810017174

**Published:** 2010-05-15

**Authors:** He-Bing Li

**Affiliations:** a Department of Chemistry and Life Sciences, Xiangnan University, Chenzhou 423000, People’s Republic of China

## Abstract

In the centrosymmetric binuclear title complex, [Cu_2_(C_13_H_19_N_2_O)_2_(N_3_)_2_], the Cu^II^ atom adopts an elongated CuON_4_ square-based pyramidal coordination geometry, arising from the *N*,*N*′,*O*-tridentate ligand and two bridging end-on azide anions. The O atom is in the basal plane, one of the azide N atoms is in the apical site and the Cu⋯Cu separation is 3.2365 (3) Å. A pair of intra­molecular N—H⋯O hydrogen bonds helps to establish the mol­ecular conformation.

## Related literature

For background to polynuclear complexes, see: Massoud *et al.* (2007 ▶); Lisnard *et al.* (2007 ▶); Sarkar *et al.* (2004 ▶); Escuer & Aromí (2006 ▶); Goher *et al.* (2001 ▶); Colacio *et al.* (2005 ▶); Sailaja *et al.* (2003 ▶); Cheng *et al.* (2006 ▶); Meyer *et al.* (2005 ▶); Sharma (1990 ▶); Ko *et al.* (2006 ▶); Escuer *et al.* (1998 ▶). For azido-bridged copper(II) complexes, see: Triki *et al.* (2005 ▶); Gao *et al.* (2005 ▶); Zhang *et al.* (2001 ▶).
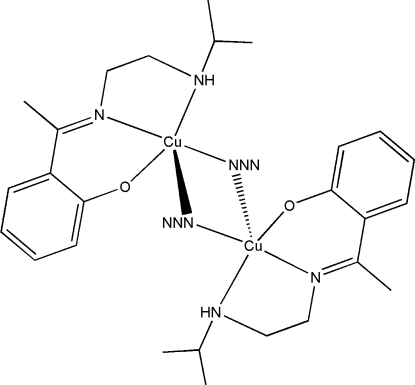


## Experimental

### 

#### Crystal data

[Cu_2_(C_13_H_19_N_2_O)_2_(N_3_)_2_]
*M* *_r_* = 649.74
Monoclinic, 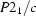

*a* = 9.6558 (3) Å
*b* = 15.3021 (5) Å
*c* = 10.6549 (3) Å
β = 115.174 (1)°
*V* = 1424.78 (8) Å^3^
*Z* = 2
Mo *K*α radiation
μ = 1.54 mm^−1^
*T* = 298 K
0.30 × 0.28 × 0.27 mm

#### Data collection

Bruker SMART CCD diffractometer
Absorption correction: multi-scan (*SADABS*; Bruker, 1998 ▶) *T*_min_ = 0.656, *T*_max_ = 0.682
8486 measured reflections
3205 independent reflections
2700 reflections with *I* > 2σ(*I*)
*R*_int_ = 0.022

#### Refinement

*R*[*F* ^2^ > 2σ(*F* ^2^)] = 0.026
*wR*(*F* ^2^) = 0.068
*S* = 1.05
3205 reflections
184 parameters
H-atom parameters constrained
Δρ_max_ = 0.21 e Å^−3^
Δρ_min_ = −0.33 e Å^−3^

### 

Data collection: *SMART* (Bruker, 1998 ▶); cell refinement: *SAINT* (Bruker, 1998 ▶); data reduction: *SAINT*; program(s) used to solve structure: *SHELXS97* (Sheldrick, 2008 ▶); program(s) used to refine structure: *SHELXL97* (Sheldrick, 2008 ▶); molecular graphics: *SHELXTL* (Sheldrick, 2008 ▶); software used to prepare material for publication: *SHELXTL*.

## Supplementary Material

Crystal structure: contains datablocks global, I. DOI: 10.1107/S1600536810017174/hb5441sup1.cif

Structure factors: contains datablocks I. DOI: 10.1107/S1600536810017174/hb5441Isup2.hkl

Additional supplementary materials:  crystallographic information; 3D view; checkCIF report

## Figures and Tables

**Table 1:** Selected bond lengths (Å)

Cu1—O1	1.8786 (13)
Cu1—N1	1.9604 (14)
Cu1—N3	2.0067 (15)
Cu1—N2	2.0369 (14)
Cu1—N3^i^	2.4175 (16)

Symmetry code: (i) 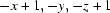.

**Table 2:** Hydrogen-bond geometry (Å, °)

*D*—H⋯*A*	*D*—H	H⋯*A*	*D*⋯*A*	*D*—H⋯*A*
N2—H2*A*⋯O1^i^	0.91	2.45	3.293 (2)	155

Symmetry code: (i) 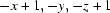.

**Fig. 1.**
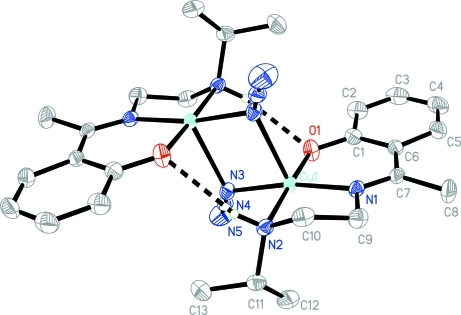
The molecular structure of (I) showing 30% probability displacement ellipsoids. The dashed lines indicate the N—H···O hydrogen bonds. Unlabelled atoms are generated by (1–x, –y, 1–z).

**Fig. 2.**
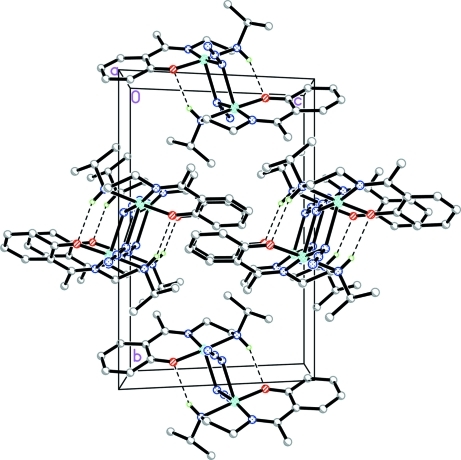
The packing diagram for (I).

## supplementary crystallographic information

### Comment

Polynuclear complexes containing bridging groups are of great interest because of their versatile molecular structures and applications (Massoud *et al.*, 2007; Lisnard *et al.*, 2007; Sarkar *et al.*, 2004). In the last few years chemists have dedicated their efforts to the study of molecular-based magnetic materials. One strategy for the design of molecular based magnets involves assembling of paramagnetic metal ions in one-, two- and three-dimensional networks using suitable bridging ligands (Escuer & Aromí, 2006; Goher *et al.*, 2001; Colacio *et al.*, 2005; Sailaja *et al.*, 2003). The azide ligands have been widely used because of their diverse binding modes that yield different types of molecules such as dimmers, tetramers, one-, two-, or three-dimensional arrays (Cheng *et al.*, 2006; Meyer *et al.*, 2005; Sharma, 1990; Ko *et al.*, 2006; Escuer *et al.*, 1998). In the present work, the title new end-on azido-bridged dinuclear copper(II) complex, (I), containing the deprotonated form of 2-[1-(2-isopropylaminoethylimino)ethyl]phenol), HL, has been prepared and structural characterized.

The structure of the complex is shown in Fig. 1. There are two unique units [CuL] linked by double end-on azido bridging groups with an inversion center at the midpoint of the two Cu atoms. Each Cu atom in the complex is in a square pyramidal environment consisting of the NNO donor set from one Schiff base ligand and two N atoms from two bridging azido groups. The Cu···Cu distance is 3.236 (1) Å. The Cu—O and Cu—N bond lengths are comparable to the corresponding values observed in other similar copper(II) complexes with azido bridges (Triki *et al.*, 2005; Gao *et al.*, 2005; Zhang *et al.*, 2001). There are two N—H···O hydrogen bonds (Table 1) between the two symmetry-related two CuL units (Fig. 2).

### Experimental

A mixture of NaN_3_ (0.065 g, 1 mmol) and Cu(NO_3_)_2_.3H_2_O (0.241 g, 1 mmol) in 50 ml methanol was stirred for half an hour with heating, then HL (0.220 g, 1 mmol) was added to the solution and the reaction continued to stirred for 1 h. After filtration, the blue filtrate was allowed to stand at room temperature for a week to deposit blue blocks of (I) in 54% yield.

### Refinement

H atoms were placed in geometrically idealized positions and allowed to ride on their parent atoms, with C—H = 0.93-0.98 Å, N—H = 0.91 Å, and with U_iso_(H) = 1.2U_eq_(C,*N*) and 1.5U_eq_(C_methyl_).

### Crystal data

**Table:**

[Cu_2_(C_13_H_19_N_2_O)_2_(N_3_)_2_]	*F*(000) = 676
*M**_r_* = 649.74	*D*_x_ = 1.515 Mg m^−^^3^
Monoclinic, *P*2_1_/*c*	Mo *K*α radiation, λ = 0.71073 Å
Hall symbol: -P 2ybc	Cell parameters from 4033 reflections
*a* = 9.6558 (3) Å	θ = 2.5–28.4°
*b* = 15.3021 (5) Å	µ = 1.54 mm^−^^1^
*c* = 10.6549 (3) Å	*T* = 298 K
β = 115.174 (1)°	Block, blue
*V* = 1424.78 (8) Å^3^	0.30 × 0.28 × 0.27 mm
*Z* = 2	

### Data collection

**Table:**

Bruker SMART CCD diffractometer	3205 independent reflections
Radiation source: fine-focus sealed tube	2700 reflections with *I* > 2σ(*I*)
graphite	*R*_int_ = 0.022
ω scans	θ_max_ = 27.5°, θ_min_ = 2.3°
Absorption correction: multi-scan (*SADABS*; Bruker, 1998)	*h* = −12→12
*T*_min_ = 0.656, *T*_max_ = 0.682	*k* = −19→15
8486 measured reflections	*l* = −13→12

### Refinement

**Table:**

Refinement on *F*^2^	Primary atom site location: structure-invariant direct methods
Least-squares matrix: full	Secondary atom site location: difference Fourier map
*R*[*F*^2^ > 2σ(*F*^2^)] = 0.026	Hydrogen site location: inferred from neighbouring sites
*wR*(*F*^2^) = 0.068	H-atom parameters constrained
*S* = 1.05	*w* = 1/[σ^2^(*F*_o_^2^) + (0.0336*P*)^2^ + 0.2887*P*] where *P* = (*F*_o_^2^ + 2*F*_c_^2^)/3
3205 reflections	(Δ/σ)_max_ = 0.001
184 parameters	Δρ_max_ = 0.21 e Å^−^^3^
0 restraints	Δρ_min_ = −0.32 e Å^−^^3^

### Special details

**Table:**

Geometry. All esds (except the esd in the dihedral angle between two l.s. planes) are estimated using the full covariance matrix. The cell esds are taken into account individually in the estimation of esds in distances, angles and torsion angles; correlations between esds in cell parameters are only used when they are defined by crystal symmetry. An approximate (isotropic) treatment of cell esds is used for estimating esds involving l.s. planes.
Refinement. Refinement of *F*^2^ against ALL reflections. The weighted *R*-factor wR and goodness of fit *S* are based on *F*^2^, conventional *R*-factors *R* are based on *F*, with *F* set to zero for negative *F*^2^. The threshold expression of *F*^2^ > σ(*F*^2^) is used only for calculating *R*-factors(gt) etc. and is not relevant to the choice of reflections for refinement. *R*-factors based on *F*^2^ are statistically about twice as large as those based on *F*, and *R*- factors based on ALL data will be even larger.

### Fractional atomic coordinates and isotropic or equivalent isotropic displacement parameters (Å^2^)

**Table:**

	*x*	*y*	*z*	*U*_iso_*/*U*_eq_	
Cu1	0.59044 (2)	0.085868 (13)	0.58341 (2)	0.02936 (8)	
N1	0.79784 (16)	0.13417 (9)	0.67367 (16)	0.0338 (3)	
N2	0.58778 (17)	0.13250 (10)	0.40334 (15)	0.0342 (3)	
H2A	0.5430	0.0905	0.3380	0.041*	
N3	0.36638 (17)	0.05814 (10)	0.48628 (16)	0.0365 (3)	
N4	0.27481 (17)	0.08176 (10)	0.52619 (17)	0.0381 (4)	
N5	0.1825 (3)	0.10262 (14)	0.5608 (3)	0.0738 (7)	
O1	0.59002 (15)	0.04762 (10)	0.75079 (13)	0.0455 (3)	
C1	0.7028 (2)	0.05405 (13)	0.87468 (19)	0.0389 (4)	
C2	0.6797 (3)	0.01235 (15)	0.9831 (2)	0.0524 (5)	
H2	0.5897	−0.0188	0.9615	0.063*	
C3	0.7854 (3)	0.01630 (16)	1.1183 (2)	0.0628 (7)	
H3	0.7662	−0.0110	1.1873	0.075*	
C4	0.9211 (4)	0.06119 (17)	1.1520 (2)	0.0704 (8)	
H4	0.9934	0.0643	1.2437	0.084*	
C5	0.9482 (3)	0.10079 (15)	1.0498 (2)	0.0559 (6)	
H5	1.0408	0.1297	1.0741	0.067*	
C6	0.8418 (2)	0.09990 (12)	0.9086 (2)	0.0386 (4)	
C7	0.8821 (2)	0.14202 (12)	0.8055 (2)	0.0373 (4)	
C8	1.0266 (2)	0.19634 (16)	0.8554 (3)	0.0606 (6)	
H8A	1.1115	0.1596	0.8663	0.091*	
H8B	1.0449	0.2227	0.9429	0.091*	
H8C	1.0150	0.2412	0.7887	0.091*	
C9	0.8460 (2)	0.17343 (14)	0.5724 (2)	0.0443 (5)	
H9A	0.9538	0.1620	0.5996	0.053*	
H9B	0.8310	0.2362	0.5691	0.053*	
C10	0.7519 (2)	0.13412 (13)	0.4315 (2)	0.0433 (5)	
H10A	0.7652	0.1683	0.3608	0.052*	
H10B	0.7868	0.0751	0.4283	0.052*	
C11	0.5038 (2)	0.21591 (13)	0.3447 (2)	0.0434 (5)	
H11	0.5600	0.2479	0.3014	0.052*	
C12	0.4930 (3)	0.27349 (15)	0.4550 (3)	0.0587 (6)	
H12A	0.4341	0.2444	0.4959	0.088*	
H12B	0.4442	0.3275	0.4141	0.088*	
H12C	0.5939	0.2852	0.5253	0.088*	
C13	0.3461 (3)	0.19476 (17)	0.2336 (2)	0.0636 (6)	
H13A	0.3555	0.1580	0.1646	0.095*	
H13B	0.2948	0.2479	0.1911	0.095*	
H13C	0.2880	0.1649	0.2745	0.095*	

### Atomic displacement parameters (Å^2^)

**Table:**

	*U*^11^	*U*^22^	*U*^33^	*U*^12^	*U*^13^	*U*^23^
Cu1	0.02708 (12)	0.03103 (13)	0.03107 (13)	−0.00587 (8)	0.01341 (9)	−0.00101 (9)
N1	0.0281 (7)	0.0312 (8)	0.0426 (9)	−0.0043 (6)	0.0154 (7)	−0.0021 (7)
N2	0.0403 (8)	0.0307 (8)	0.0337 (8)	−0.0058 (6)	0.0177 (7)	−0.0011 (6)
N3	0.0299 (8)	0.0390 (8)	0.0417 (9)	−0.0062 (6)	0.0163 (7)	−0.0048 (7)
N4	0.0319 (8)	0.0322 (8)	0.0478 (9)	−0.0015 (6)	0.0147 (7)	0.0014 (7)
N5	0.0586 (13)	0.0661 (14)	0.117 (2)	0.0054 (10)	0.0569 (14)	−0.0125 (13)
O1	0.0376 (7)	0.0671 (9)	0.0331 (7)	−0.0116 (7)	0.0163 (6)	0.0023 (7)
C1	0.0445 (10)	0.0405 (10)	0.0342 (10)	0.0072 (8)	0.0194 (8)	−0.0030 (8)
C2	0.0688 (14)	0.0548 (13)	0.0416 (12)	0.0089 (11)	0.0312 (11)	0.0021 (10)
C3	0.098 (2)	0.0558 (14)	0.0383 (12)	0.0221 (14)	0.0321 (13)	0.0041 (11)
C4	0.096 (2)	0.0579 (15)	0.0327 (12)	0.0248 (15)	0.0037 (12)	−0.0022 (11)
C5	0.0571 (13)	0.0470 (13)	0.0443 (12)	0.0090 (10)	0.0031 (10)	−0.0091 (10)
C6	0.0387 (10)	0.0325 (10)	0.0367 (10)	0.0073 (8)	0.0085 (8)	−0.0078 (8)
C7	0.0292 (9)	0.0293 (9)	0.0470 (11)	0.0014 (7)	0.0100 (8)	−0.0078 (8)
C8	0.0366 (11)	0.0606 (15)	0.0692 (15)	−0.0137 (10)	0.0077 (10)	−0.0124 (13)
C9	0.0340 (10)	0.0451 (11)	0.0589 (13)	−0.0066 (8)	0.0248 (9)	0.0039 (10)
C10	0.0486 (11)	0.0415 (11)	0.0541 (12)	−0.0014 (9)	0.0356 (10)	0.0051 (9)
C11	0.0492 (11)	0.0359 (10)	0.0447 (11)	−0.0013 (8)	0.0195 (9)	0.0110 (9)
C12	0.0685 (15)	0.0389 (12)	0.0668 (15)	0.0079 (11)	0.0272 (13)	−0.0025 (11)
C13	0.0588 (14)	0.0623 (16)	0.0517 (14)	0.0021 (12)	0.0062 (11)	0.0125 (12)

### Geometric parameters (Å, °)

**Table:**

Cu1—O1	1.8786 (13)	C5—C6	1.416 (3)
Cu1—N1	1.9604 (14)	C5—H5	0.9300
Cu1—N3	2.0067 (15)	C6—C7	1.462 (3)
Cu1—N2	2.0369 (14)	C7—C8	1.513 (3)
Cu1—N3^i^	2.4175 (16)	C8—H8A	0.9600
N1—C7	1.295 (2)	C8—H8B	0.9600
N1—C9	1.473 (2)	C8—H8C	0.9600
N2—C10	1.482 (2)	C9—C10	1.510 (3)
N2—C11	1.499 (2)	C9—H9A	0.9700
N2—H2A	0.9100	C9—H9B	0.9700
N3—N4	1.189 (2)	C10—H10A	0.9700
N3—Cu1^i^	2.4175 (16)	C10—H10B	0.9700
N4—N5	1.145 (2)	C11—C12	1.507 (3)
O1—C1	1.310 (2)	C11—C13	1.514 (3)
C1—C2	1.417 (3)	C11—H11	0.9800
C1—C6	1.418 (3)	C12—H12A	0.9600
C2—C3	1.368 (3)	C12—H12B	0.9600
C2—H2	0.9300	C12—H12C	0.9600
C3—C4	1.384 (4)	C13—H13A	0.9600
C3—H3	0.9300	C13—H13B	0.9600
C4—C5	1.364 (4)	C13—H13C	0.9600
C4—H4	0.9300		
			
O1—Cu1—N1	93.78 (6)	C1—C6—C7	123.53 (17)
O1—Cu1—N3	89.23 (6)	N1—C7—C6	121.92 (16)
N1—Cu1—N3	170.06 (6)	N1—C7—C8	119.49 (18)
O1—Cu1—N2	177.52 (6)	C6—C7—C8	118.59 (18)
N1—Cu1—N2	86.04 (6)	C7—C8—H8A	109.5
N3—Cu1—N2	90.54 (6)	C7—C8—H8B	109.5
O1—Cu1—N3^i^	94.47 (6)	H8A—C8—H8B	109.5
N1—Cu1—N3^i^	102.75 (5)	C7—C8—H8C	109.5
N3—Cu1—N3^i^	86.43 (6)	H8A—C8—H8C	109.5
N2—Cu1—N3^i^	87.98 (6)	H8B—C8—H8C	109.5
C7—N1—C9	120.54 (15)	N1—C9—C10	108.74 (15)
C7—N1—Cu1	127.34 (13)	N1—C9—H9A	109.9
C9—N1—Cu1	111.69 (12)	C10—C9—H9A	109.9
C10—N2—C11	114.37 (14)	N1—C9—H9B	109.9
C10—N2—Cu1	103.35 (11)	C10—C9—H9B	109.9
C11—N2—Cu1	118.59 (11)	H9A—C9—H9B	108.3
C10—N2—H2A	106.6	N2—C10—C9	110.37 (15)
C11—N2—H2A	106.6	N2—C10—H10A	109.6
Cu1—N2—H2A	106.6	C9—C10—H10A	109.6
N4—N3—Cu1	123.64 (13)	N2—C10—H10B	109.6
N4—N3—Cu1^i^	129.69 (12)	C9—C10—H10B	109.6
Cu1—N3—Cu1^i^	93.57 (6)	H10A—C10—H10B	108.1
N5—N4—N3	177.4 (2)	N2—C11—C12	112.19 (16)
C1—O1—Cu1	126.66 (12)	N2—C11—C13	109.26 (17)
O1—C1—C2	115.87 (18)	C12—C11—C13	110.81 (19)
O1—C1—C6	125.78 (17)	N2—C11—H11	108.2
C2—C1—C6	118.34 (19)	C12—C11—H11	108.2
C3—C2—C1	122.2 (2)	C13—C11—H11	108.2
C3—C2—H2	118.9	C11—C12—H12A	109.5
C1—C2—H2	118.9	C11—C12—H12B	109.5
C2—C3—C4	119.6 (2)	H12A—C12—H12B	109.5
C2—C3—H3	120.2	C11—C12—H12C	109.5
C4—C3—H3	120.2	H12A—C12—H12C	109.5
C5—C4—C3	119.7 (2)	H12B—C12—H12C	109.5
C5—C4—H4	120.2	C11—C13—H13A	109.5
C3—C4—H4	120.2	C11—C13—H13B	109.5
C4—C5—C6	123.0 (2)	H13A—C13—H13B	109.5
C4—C5—H5	118.5	C11—C13—H13C	109.5
C6—C5—H5	118.5	H13A—C13—H13C	109.5
C5—C6—C1	117.1 (2)	H13B—C13—H13C	109.5
C5—C6—C7	119.31 (19)		

Symmetry codes: (i) −*x*+1, −*y*, −*z*+1.

### Hydrogen-bond geometry (Å, °)

**Table:**

*D*—H···*A*	*D*—H	H···*A*	*D*···*A*	*D*—H···*A*
N2—H2A···O1^i^	0.91	2.45	3.293 (2)	155

Symmetry codes: (i) −*x*+1, −*y*, −*z*+1.
